# Impact of dephased entanglement, mixed input states and varying measurement orientations on the reliability of cryptographic keys generated via the quantum protocol E91

**DOI:** 10.1371/journal.pone.0360669

**Published:** 2026-10-21

**Authors:** Adrián F. Hernández-Borda, María P. Rojas-Sepúlveda, Hanz Y. Ramírez-Gómez

**Affiliations:** Grupo QUCIT, Escuela de Física, Universidad Pedagógica y Tecnológica de Colombia, Tunja, Colombia; University of Florence: Universita degli Studi di Firenze, ITALY

## Abstract

Photons and optical circuits are among the most promising platforms for implementation of quantum technologies, including quantum computing and quantum cryptography. For entanglement-based quantum key distribution, on-demand sources of highly entangled photon pairs are essential, and semiconductor quantum dots have emerged as prominent candidates to satisfy the necessary conditions of brightness and fidelity. However, in most cases the photon pair emitted by these sources is an anomalous Bell state whose relative phase and purity are non-ideal, and then, their suitability for distributing secret keys must be assessed. This work studies the performance of the quantum key distribution protocol E91, under the influence of three elements: the phase in the input state, the impurity of such a state, and the relative directions of the polarization analyzers. We derive analytical expressions for the minimum quantum bit error rate and the Bell’s parameter, and validate them by using quantum computational tools. Our results show that the performance of the quantum transmission is highly impacted by the phase and purity of the entangled input states, and that such an impact may be modulated through the orientation of the polarizers. These findings provide important insight for the scalable implementation of quantum key distribution protocols with realistic entanglement sources.

## 1 Introduction

Quantum key distribution (QKD) is a private-key cryptographic model that exploits the principles of quantum mechanics such as superposition, entanglement, and collapse of quantum states, to transmit the constituent bits of secure keys for encryption and decryption of information [1–4]. The superiority of quantum protocols as compared to classical counterparts for the secure exchange of sensitive information in networks, has been widely argued and theoretically demonstrated, albeit the technology for their large-scale implementation is still in development [5,6].

Realization of this type of cryptographic schemes has been achieved mainly with the use of optical quantum entangled states, what has promoted the photonic implementation as a viable option for quantum communication and quantum internet [7–12]. Hence, the development of optimized entangled-photon sources has become a topic of intense research toward the progress of light-based quantum technologies [13–16].

Quantum entangled states were subject of controversy and intensively discussed during the second part of the XX century, since their introduction by Einstein, Podolsky, and Rosen [17]. Intending to resolve the dispute on the reality of those perplexing states, J. Bell mathematically proved that any hidden variable theory is incompatible with the statistical predictions of quantum mechanics [18]. This result is known as the Bell’s Theorem, on which subsequently other authors elaborated to propose their own versions of the theorem, generating a set of expression named Bell inequalities. Particularly, Clauser et al. in reference [19], developed an expression known as the CHSH inequality. This inequality has been experimentally verified via photon-based experiments in which, first, a radiative cascade decay in calcium atoms, and later, spontaneous parametric down conversion (SPDC) in nonlinear crystals, were used as entangled-photon sources [20–23].

SPDC has been so far the most commonly employed mechanism for entanglement generation, because of the high fidelity of entangled states obtained by this method [24,25]. However, its production of photon pairs is random and then unsuitable for reliable quantum communication or other applications that require an on-demand source of entangled states [26,27]. In this scenario, quantum dots (QDs) have appeared as promising candidates for on-demand generation of entanglement. In fact, significant progress has been reported in the use of QDs as sources of non-classical states of light for applications in quantum information and quantum communications [10,28–35].

Nonetheless, QD-produced entangled states are frequently dephased with respect to the ideal Bell states for which the QKD protocols are usually designed. Such a dephasing is underlaid by the so-called electron-hole exchange, that causes the exciton fine structure splitting (FSS) in strongly confined nanostructures [36–40].

The so-called E91 protocol was the first quantum entanglement-based QKD protocol, devised by A. K. Ekert in the early 90’s [2,41]. It includes a mechanism for validating the security of the transmitted key based on measurements of Bell inequalities, which allows to rule out eavesdropping. That verification is normally carried out by means of the CHSH inequality [23,42].

In the original version of this protocol, the author proposed the use of 1/2-spin particles prepared in a Bell’s state. Nevertheless, at the last part of that seminal work, he mentioned that an optimal realization could be based on correlated photon states.

Indeed, this QKD scheme was first effectively implemented by using polarization-entangled photon states produced via SPDC [8,43]. Afterwards, entangled photons obtained from semiconductor quantum dots were used, achieving long-distance transmission and acceptable fidelity, although the yielded brightness (and then the secure key rate) was found below what could be achieved with a SPDC source [30,44]. This behavior can be related to spectral filtering or to reduction of the electron-hole overlap by electrical driving applied to suppress the FSS [36,45,46]. Then, an important trade-off is posed: elimination of the relative phase in the emitted pairs reduces the brightness of the source, while bright QD sources would emit dephased entangled states whose noisy effects are so far, mostly undetermined.

Improvements in the implementation of QKD protocols toward industrial use, require clear understanding of the influence of different noise channels on their performance and reliability. In the case of entanglement-based protocols, three phases can be identified: i) Generation of the entangled pairs, ii) Transmission of the entangled qubits, and iii) Execution of the protocol itself (measurements, sifting and validation).

Noise effects on the efficacy of QKD protocols have been recently reported [39,47–51]. In particular, a couple of relevant studies have been devoted to the notorious protocol E91.

In the first one, L. Mariani et al. simulated the influence of qubit depolarization and non-ideal detection, which correspond to phases ii) and iii) in our description above [52]. On the other hand, H. Alhashim in reference [53], studied the consequences of attenuated transmission and entanglement decoherence, which are different perturbation channels as compared to Mariani’s work, but are also mainly related to phase ii). Both of them demonstrate significant dependence of the quantum bit error rate on the level of disturbance associated to the considered flaws. However, those works assume ideal input states, although availability of such a faultless generator is unlikely.

Actually, imperfection of the input entangled states is the earliest and one of the most significant noise mechanisms in the implementation of entanglement-based QKD protocols. Thus, any comprehensive understanding of noise effects on their performance is incomplete without characterization of the sequels associated to realistic sources of input states.

In this work we study the effects of the main noise channels in phase i) of the E91 protocol: The FSS-driven dephasing in the entangled input state and the impurity of the two-photon emitted state. We additionally study the influence of the relative orientation between measurement axes on the performance of this QKD scheme. We address the problem with the aid of a quantum computing simulator, which mimics the corresponding laborious optical experiment with a succinct implementation in the IBM’s API Qiskit. In the first part we introduce the dephasing effects of the FSS on the entangled states, and impurity of the photon pairs produced in QDs. Afterward, we explore the interplay between non-ideal input states and the orientation of detection axes in the execution of the QKD protocol E91, and derive the corresponding analytical expressions. Finally, we validate our model by means of the quantum computing implementation and discuss the impact of the analyzed variables on the performance of the key distribution process.

## 2 Sources of entangled photon pairs for QKD

Many of the fundamental experiments that have provided verification of the physical reality behind entanglement, as well as most of the QKD experiments carried out to date, have used SPDC to produce quantum correlated photons [7–9,54,55]. This mechanism permits the creation of a pair of lower-energy daughter photons originated from a higher-energy pump photon. That triggering photon interacts with a transparent non-linear crystal in such a way that a polarization-entangled photon pair may or may not be generated [56]. Because SPDC is not an on-demand process, its use for implementations of scaled quantum communication is dubious. The probability of obtaining entangled pairs given an incident photon, is described by a Poissonian distribution. Hence, probabilistically none, one or several pairs of entangled photons may be produced, being none much more likely under normal conditions than the other components. Thus, the efficiency of this entanglement-generation method is very low and its potential for applications in emerging quantum technologies is clearly limited [57–59].

As adequate substitutes for those stochastic sources, QDs have been proposed as a deterministic on-demand source of highly entangled photon pairs by exploiting the recombination of the biexciton state (|XX⟩). This process yields two polarization-entangled photons that, in contrast to photons produced by SPDC, can be successfully applied in quantum communication processes [28,37,46,60,61].

Nevertheless, the coherence and purity of the entangled state generated in a QD, may be altered as a result of the interactions between the produced photons and its environment, such as recapture or re-population via defects; or as consequence of the FSS associated to intrinsic characteristics of the emitting dot. In particular, this latter effect has been widely studied and identified as the main challenge toward reliable generation of entangled states from QDs [46,62,63].

### 2.1 Entangled photon pairs from radiative cascades in QDs

The process starts with excitation of the neutral biexciton state |XX⟩, that later decays into one of the single exciton states |X⟩ by one of two possible decay routes (either to |X,−1⟩ and |X,1⟩). Through the first pathway, a photon with right circular polarization $\left|R_{XX}\right\rangle$ is emitted and the system’s *z*-component of angular momentum passes from *m* = 0 to m=−1 (decaying to the state |X,−1⟩). Contrarily, through the second pathway, the emitted photon has left circular polarization $\left|L_{XX}\right\rangle$ and the system passes from *m* = 0 to *m* = 1 (decaying to the state |X,1⟩). Afterwards, the corresponding exciton state decays into the ground state (|0⟩) emitting a photon with opposite polarization respect to the previously emitted one, and the system returns to *z*-component of total angular momentum *m* = 0 [37,64]. This radiative cascade and its two pathways are depicted in Fig 1, where cyan (orange) represents emission of a right (left) circularly polarized photon and *S* stands for the FSS energy.

**Fig 1:**
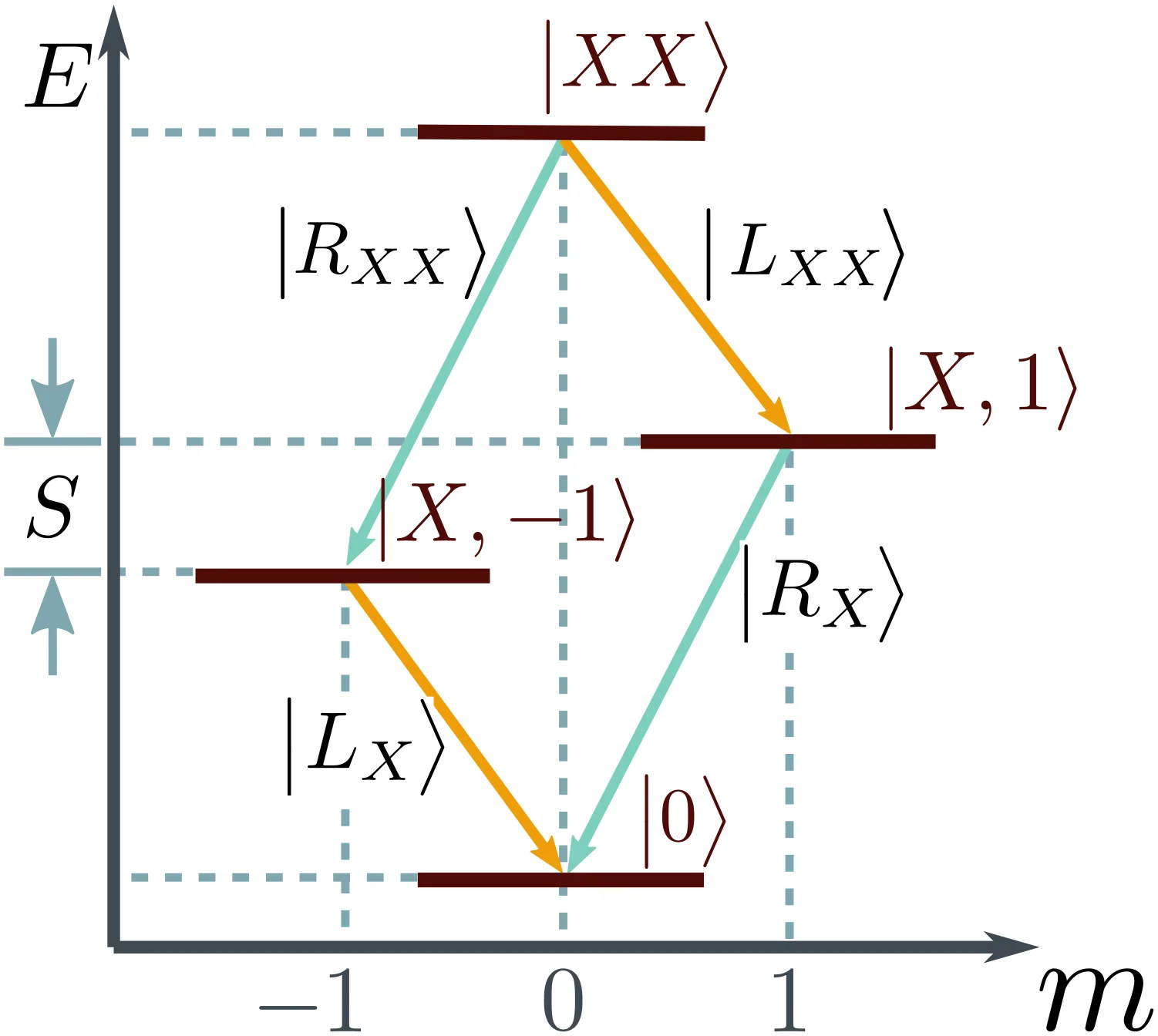
Radiative cascade |XX⟩→|X⟩→|0⟩ in a quantum dot. There are two different decay routes, one through state |X,−1⟩ (m=0→m=−1→m=0) and the other one through state |X,1⟩ (m=0→m=1→m=0). The cyan (orange) line represents emission of a right (left) circularly polarized photon.

In the case *S* = 0, the two decay pathways are indistinguishable. However, if S≠0 the degeneracy of exciton states with different angular momentum is lifted. This makes the decay routes distinguishable and the two-photon entangled state becomes


$$\begin{array}{c} |\psi \rangle =\frac{1}{\sqrt{2}}\left[|L_{XX}R_{X}\rangle +e^{-i\theta_{\text{FSS}}}|R_{XX}L_{X}\rangle \right], \end{array}$$ (1)

where the relative phase $\theta_{\text{FSS}}=S\tau /\hbar$, depends on the splitting energy *S* and on the time between the first and second electron-hole recombination τ (neutral exciton radiative lifetime) [37,47,65]. Then, the dephasing introduced by the FSS into the entangled output state, is a direct consequence of the energy splitting between the exciton states (|X,−1⟩ and |X,1⟩) [39,60,61,66].

This state is maximally entangled disregarding the value of $\theta_{\text{FSS}}$, because the relative phase does not affect the Von Neumann entropy of the system. However, such a dephasing creates an oscillation between Bell states along time. In other words, the electron-hole exchange induces via the FSS, a local unitary transformation over the Bell singlet state in the Poincare’s sphere that affects its stationary character [39,67,68].

The state in equation 1 can be rewritten in terms of linear horizontal (H) and vertical (V) polarizations, according to


$$\begin{array}{c} |\psi \rangle \;=\;\frac{1}{\sqrt{2}}\left[|H_{XX}H_{X}\rangle +e^{-i\theta_{\text{FSS}}}|V_{XX}V_{X}\rangle \right]. \end{array}$$ (2)

Tuning of the FSS in QDs has been a field of intense research, since the related dephasing produces time dependent oscillations in the fidelity, hurting the possibility of determining the degree of entanglement of the emitted states [69].

Diverse ways of reducing the energy difference *S* in QDs have been proposed, aiming to reshape the wave function of the confined carriers, to compensate the asymmetries that strengthen the magnitude of the electron-hole exchange interaction [46,70–73].

Although successful QD tuning has been realized using either piezoelectric substrates or stark effect with external electric fields [15,60,74–76], still most grown QD samples exhibit non-vanishing FSS due to the inherent lack of rotational symmetry associated to both, the microscopic crystalline structures and the non-perfectly axial dot shapes. Furthermore, reduction of the FSS may imply reduction of the source’s brightness, via weakening of the oscillator strength in the radiative dipole [36,40]

### 2.2 Mixed photon pairs from QD sources

Beside the relative phase in the entangled pairs, there is the fact that photon pairs emitted from QDs may not result from the radiative cascade but from re-excitation processes, thus affecting the purity of the entangled input states [77,78].

It has been reported that the second main production channel of photon pairs in QD sources, is the reexcitation of the biexciton state [79–81]. It occurs when either the pumping laser or the initially emitted photon re-populates the |X⟩ state immediately after the emission from the |XX⟩ state, and then the radiative cascade is interrupted [82,83].

To describe the photon pair emitted by the QD source as an ensemble of states, the density matrix formalism is employed. Let us assume the source emits the targeted entangled state, whose pure state density operator is $\rho_{\text{ent}}=\left|\psi \right\rangle \left\langle \psi \right|$ (with |ψ⟩ given by Equation 2), alongside with some other undesired states $\left|\varphi_{\ell }\right\rangle$ (ℓ=1,2,...). The resulting mixed assemble is given by the operator


$$\begin{array}{c} \rho =P_{\text{ent}}\left|\psi \right\rangle \left\langle \psi \right|+\sum_{\ell }P_{\ell }\left|\varphi_{\ell }\right\rangle \left\langle \varphi_{\ell }\right| \end{array}$$ (3)

where *P*_ent_ is the probability of obtaining the entangled state, while $P_{\ell }\ll P_{\text{ent}}$ (ℓ=1,2,...) represents the probability of measuring any of the additional parasitic states.

Those additional states considered in the ensemble ($\left|\varphi_{\ell }\right\rangle$), are included in agreement with the measurements on photon pairs emitted by QD sources described in reference [15]. Namely, such states are those of the basis $\left\{\left|H_{XX}H_{X}\right\rangle ,\left|H_{XX}V_{X}\right\rangle ,\left|V_{XX}H_{X}\right\rangle ,\left|V_{XX}V_{X}\right\rangle \right\}$, which are obviously separable.

The experimental reconstruction of the density matrix reported there, can be achieved by mapping the polarization measurements onto the same two-photon polarization basis [84], and then, the value of *P*_ent_ can be determined by means of the expression


$$\begin{array}{c} P_{\text{ent}}=1-\sum_{\ell =1}^{4}P_{\ell }. \end{array}$$ (4)

Because the re-excitation mechanism generates uncorrelated photon pairs at roughly equal rates across all basis states, if we would approximate the emission of undesired states as uniform, $P_{\text{ent}}\approx 1-4{\bar{P}}_{\text{mix}}$ would hold, where ${\bar{P}}_{\text{mix}}$ would be the averaged probability of measuring one of the parasitic states. That emission probability for non-correlated photon pairs is directly related to the second order correlation function *g*^(2)^(0) and the duration of the laser pulse [78,82].

The FSS as well as other minor sources of decoherence might produce dephasing in the density operator of the emitted pair, they would mainly affect off-diagonal elements of the corresponding matrix, maintaining the probabilities *P*_ent_ and $P_{\ell }$ mostly unaffected.

## 3 QKD protocol E91

The original E91 protocol aims the communication of a private key between two distant subjects, Alice and Bob, encoded on the spin of a pair of entangled 1/2-spin particles prepared on a singlet state. However, Ekert ended his proposal suggesting that an optical implementation could be more suitable [2]. Such implementation with photons was actually realized around a year later as reported in reference [8].

The protocol begins with the preparation of a polarization-entangled Bell state, then one photon is sent to Alice and the other one to Bob. Each participant is expected to measure his/her corresponding particle with adjustable polarizers. Each apparatus (Alice’s and Bob’s) can be set in three possible directions that are part of a group of four preset orientations, defined by its angle with respect to the vertical axis. These orientations are labeled $\phi_{i}$, *i* = 0,1,2,3, and their angles in the photonic version of the original protocol are defined according to $\phi_{\ell }=\ell \pi /8$ for ℓ=0,1,2,3, as illustrated in Fig 2(a).

**Fig 2:**
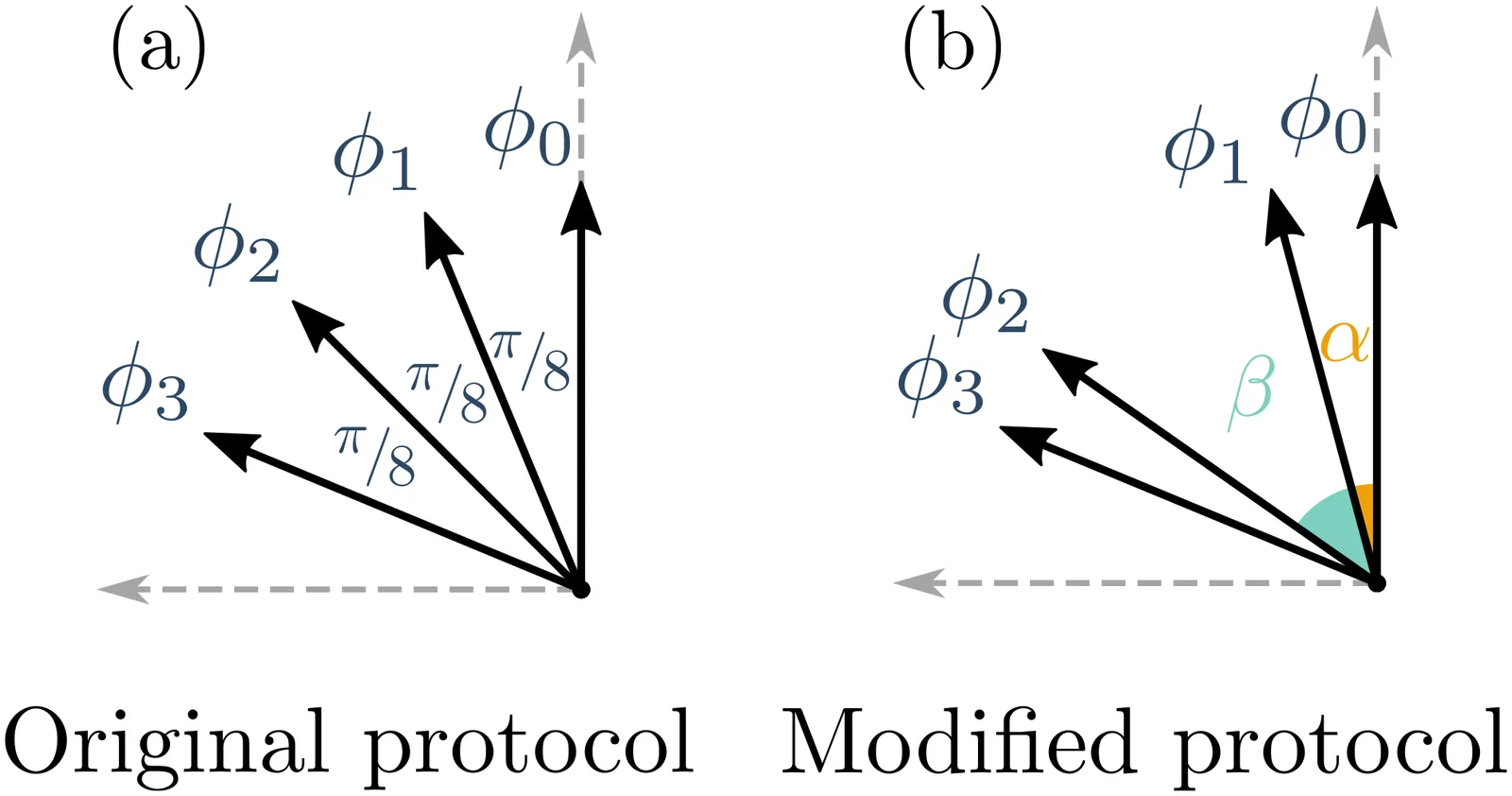
Angles for the optical implementation of the E91 protocol. (a) Fixed measurement directions in the originally proposed version of the protocol. (b) Variable measurement directions in the modified version of the protocol.

Afterwards, Alice randomly selects one of the first three directions and registers her choice ($\phi_{i_{A}}$). Then, measures the particle and records either −1 or 1, depending on the result of her measurement. In turn, Bob selects one of the last three directions, and also records his chosen orientation ($\phi_{i_{B}}$) and the corresponding measurement.

In this form, the second and third directions among the set of four ($\phi_{1}=\pi /8$ and $\phi_{2}=\pi /4$), are part of the Alice’s and Bob’s possibilities, and then, the ones in which there can be coincidence. Once the transmission has concluded, each participant shares on a public channel his/her list with the selected orientations for each event. Each participant analyzes the other’s list and compares it with his/her own to separate his/her registered measurements in two groups: the first contains the measurements for which Alice and Bob chose the same direction, and the second includes the ones taken along mismatched orientations. The cryptographic key is the string of results in the first group, while the second group of measurements is employed to validate the security of the key distribution via a Bell’s test by means of the CHSH inequality.

Those particular angles defined as multiples of π/8 were intentionally picked to maximize the quantity


$$\begin{array}{c} CR=E(\phi_{0},\phi_{1})-E(\phi_{0},\phi_{3})+E(\phi_{2},\phi_{1})+E(\phi_{2},\phi_{3}), \end{array}$$ (5)

given in terms of the correlation amplitudes


$$\begin{array}{c} E(\phi_{a},\phi_{b})=-\cos\left[2(\phi_{a}-\phi_{b})\right]. \end{array}$$ (6)

Such a quantity (*CR*) is used for the Bell’s test, carried out in the validation stage of the protocol when eavesdropping is considered. For those specifically chosen angles $CR=-2\sqrt{2}$ [2,8,19,68].

### 3.1 Protocol E91 modified

We now consider a polarization-entangled state of photons produced via a QD radiative cascade, as described in section 2. Additionally, we include a generalization by defining the detection orientations $\phi_{1}$ and $\phi_{2}$ in terms of variable angles.

Hence, we introduce the parameter α, as the angle between the vertical axis and the second orientation in the set of four ($\phi_{1}$). Similarly, we define the parameter β as the angle between the two coincident directions ($\phi_{2}-\phi_{1}$). These parameters are highlighted respectively in cyan and orange, in Fig 2(b).

### 3.2 Analytical results for the correlation probabilities

#### 3.2.1 Pure input states.

Let’s assume pure entangled input states, alongside ideal detectors and operations defining the basis for measurements (ideal photon counters and error-free determination of the directions for the polarizers in the optical implementation).

If we consider two bases, i.e., the vertical-horizontal and the ϕ-rotated-ϕ-antirotated linear polarizations, for a Bell state of polarization-entangled photons $\frac{1}{\sqrt{2}}\left[|H_{XX}H_{X}\rangle +|V_{XX}V_{X}\rangle \right]$ ($\theta_{\text{FSS}}=0$), measurements on both photons are certainly correlated if they are obtained in the same basis (along the same orientation), disregarding the angle ϕ between those bases [85].

However, if the dephasing associated to the FSS is included ($\theta_{\text{FSS}}\neq 0$), the probability of correlation will depend on the rotation angle, according to


$$\begin{array}{c} P_{\pm \pm }(\phi ,\theta_{\text{FSS}})=\frac{1}{2}[C_{\phi }^{4}+S_{\phi }^{4}+2S_{\phi }^{2}C_{\phi }^{2}C_{\theta_{\text{FSS}}}], \end{array}$$ (7)

where $S_{\phi }=\sin(\phi )$ and $C_{\phi }=\cos(\phi )$. $P_{\pm \pm }$ stands for the probability of obtaining either ${\;}_{1}^{1}$ or ${\;}_{-1}^{-1}$ in both (Alice’s and Bob’s) measurements. The detailed derivation is provided in section 6 of the supporting information.

The probability of equation 7 is essential to model the performance of the QKD protocol under the effects of the exciton FSS in the QD source. This expression can be now used to theoretically evaluate the total probability of getting correlated measurements in an event in which the bases chosen by Alice and Bob coincide, even if none of those bases correspond to the vertical-horizontal one. The derived formula is


$$\begin{array}{c} P_{\text{Corr}}^{T}=\frac{1}{2}[C_{\alpha }^{4}+S_{\alpha }^{4}+2S_{\alpha }^{2}C_{\alpha }^{2}C_{\theta_{\text{FSS}}}+\;C_{\alpha +\beta }^{4}+S_{\alpha +\beta }^{4}+2S_{\alpha +\beta }^{2}C_{\alpha +\beta }^{2}C_{\theta_{\text{FSS}}}], \end{array}$$ (8)

where $P_{\text{Corr}}^{T}$ denotes the total probability of correlation. Further details are given in section 6 of the supporting information.

This expression allows to straightforwardly predict the effects of both, the FSS in the entanglement’s source and the directions of the coincident detectors, on the performance of the considered QKD protocol.

It is important to note that according to this result, while the angles α and β are irrelevant in the case $\theta_{\text{FSS}}=0$, they become determining on the effectiveness of the protocol when the FSS is not negligible.

Regarding the Bell quantity (*CR*), it is not a fixed value but rather a function that depends on α, β and $\theta_{\text{FSS}}$. It can be expressed as


$$\begin{array}{cc} CR= & C_{\theta_{\text{FSS}}}S_{2(\alpha +\beta )}\left[\frac{1}{\sqrt{2}}+S_{2\alpha }\right]+C_{2(\alpha +\beta )}\left[C_{2\alpha }-\frac{1}{\sqrt{2}}\right]+C_{2\alpha }+\frac{1}{\sqrt{2}}. \end{array}$$ (9)

The details of the derivation are shown in section 6 of the supporting information.

For the orientations originally proposed by Ekert (in the optical implementation), the equation 9 reduces to


$$\begin{array}{c} \left|CR\right|=\sqrt{2}\left|C_{\theta_{\text{FSS}}}+1\right|\leq 2\sqrt{2}. \end{array}$$ (10)

Thus, although *CR* is still bounded by $\pm 2\sqrt{2}$, it oscillates as $\theta_{\text{FSS}}$ increases, exhibiting minima (|*CR*| = 0) for $\theta_{\text{FSS}}=(2n+1)\pi$ (*n* integer). Interactive Fig 5 (a) and 5 (b) in the supporting information ease visualization of the functions given in equations 8 and 9.

#### 3.2.2 Mixed input states.

As mentioned in section 2.2, although most of the photon pairs emitted by a QD adequately excited in the state |XX⟩ are entangled, it is not realistic to assume that all of them are generated by the radiative cascade decay [15,79–81]. Thus, some of the emitted pairs cannot be written as those in equation 1, but in equation 3 and then, equations 8 and 9 are modified according to


$$\begin{array}{cc} P_{\text{Corr}}^{M} & =P_{\text{ent}}\frac{1}{2}[C_{\alpha }^{4}+S_{\alpha }^{4}+2S_{\alpha }^{2}C_{\alpha }^{2}C_{\theta_{\text{FSS}}}+\;C_{\alpha +\beta }^{4}+S_{\alpha +\beta }^{4}+2S_{\alpha +\beta }^{2}C_{\alpha +\beta }^{2}C_{\theta_{\text{FSS}}}]+\\ & \;\frac{P_{HH}+P_{VV}}{2}[C_{\alpha }^{4}+S_{\alpha }^{4}+C_{\alpha +\beta }^{4}+S_{\alpha +\beta }^{4}]+\;(P_{HV}+P_{VH})[C_{\alpha }^{2}S_{\alpha }^{2}+C_{\alpha +\beta }^{2}S_{\alpha +\beta }^{2}], \end{array}$$ (11)

and


$$\begin{array}{cc} CR^{M}= & P_{\text{ent}}\left(C_{\theta_{\text{FSS}}}S_{2(\alpha +\beta )}\left[\frac{1}{\sqrt{2}}+S_{2\alpha }\right]+C_{2(\alpha +\beta )}\left[C_{2\alpha }-\frac{1}{\sqrt{2}}\right]+C_{2\alpha }+\frac{1}{\sqrt{2}}\right)\\ & +\left(P_{HH}+P_{VV}-P_{HV}-P_{VH}\right)\left(C_{2(\alpha +\beta )}\left[C_{2\alpha }-\frac{1}{\sqrt{2}}\right]+C_{2\alpha }+\frac{1}{\sqrt{2}}\right). \end{array}$$ (12)

Equation 10, which considers the original Ekert’s directions in the optical implementation, transforms into


$$\begin{array}{c} \left|CR^{M}\right|=\sqrt{2}|P_{\text{ent}}\left(1+C_{\theta_{\text{FSS}}}\right)+P_{HH}+P_{VV}-P_{HV}-P_{VH}|\leq 2\sqrt{2}. \end{array}$$ (13)

It is worth remarking that equations 11, 12 and 13 recover their counterparts (8, 9 and 10), in the limit $P_{\ell }\to 0$ and $P_{\text{ent}}\to 1$.

Derivations of these expressions are straightforwardly similar to the ones for pure states, but including the additional components with their corresponding probabilities.

Interactive Fig 5 (c) and 5 (d) in the supporting information ease visualization of the functions given in equations 11 and 12.

## 4 Quantum computational validation

To test the validity of equations 8 and 11, instead of carrying out the delicate and grueling optical experiment, one may opt for a quantum computational implementation that emulates the QKD through the considered protocol [47].

To build a quantum circuit that emulates the quantum transmission of the key, we first encoded the polarization states into the qubit representation by using the computational basis and rotation gates, as shown in Table 1.

**Table 1:** Photon information encoded on quantum computation language.

Scheme	Unrotated Basis	Rotated Basis
PhotonPolarization	{|H⟩,|V⟩}	$\left\{e^{i\phi_{\ell }\hat{Y}/2}\left|H\right\rangle ,e^{i\phi_{\ell }\hat{Y}/2}\left|V\right\rangle \right\}$
QuantumComputation	{|0⟩,|1⟩}	$\left\{{\hat{R}}_{y}(\phi_{\ell })\left|0\right\rangle ,{\hat{R}}_{y}(\phi_{\ell })\left|1\right\rangle \right\}$

To reach this goal, we rewrite the dephased singlet state in the computational basis for two qubits, generated by the operators ${\hat{H}}_{0}⊗{\hat{I}}_{1}$ and ${\hat{CX}}_{0,1}$, where the parameterized relative phase is added by applying the gate ${\hat{R}}_{z,i}$ over any of the two qubits (either *i* = 0 or *i* = 1) [47]. Thus, the state of equation 2 reads


$$\begin{array}{c} |\psi \rangle \;=\;\frac{1}{\sqrt{2}}\left[|00\rangle +e^{-i\theta_{\text{FSS}}}|11\rangle \right]. \end{array}$$ (14)

### 4.1 Quantum circuit

Once the encoding is defined, we focus on creating a quantum circuit that: First, recreates the transmission of the corresponding input state. Second, mimics the process of basis selection performed by Alice and Bob, and saves the chosen direction. Third, it measures the quantum channels and stores the values registered by each participant.

#### 4.1.1 Circuit for pure input states.

We will begin assuming an input pure entangled state as the one shown in equation 14.

The quantum circuit implemented to emulate the generation and transmission of one key-bit is depicted in Fig 3. There, the quantum gates H, X, RY, RZ and $\dot{⊕}$, respectively represent the Hadamard, Pauli_*X*_, *Y*-rotation, *Z*-rotation and Controlled-*X* unitary operations.

**Fig 3:**
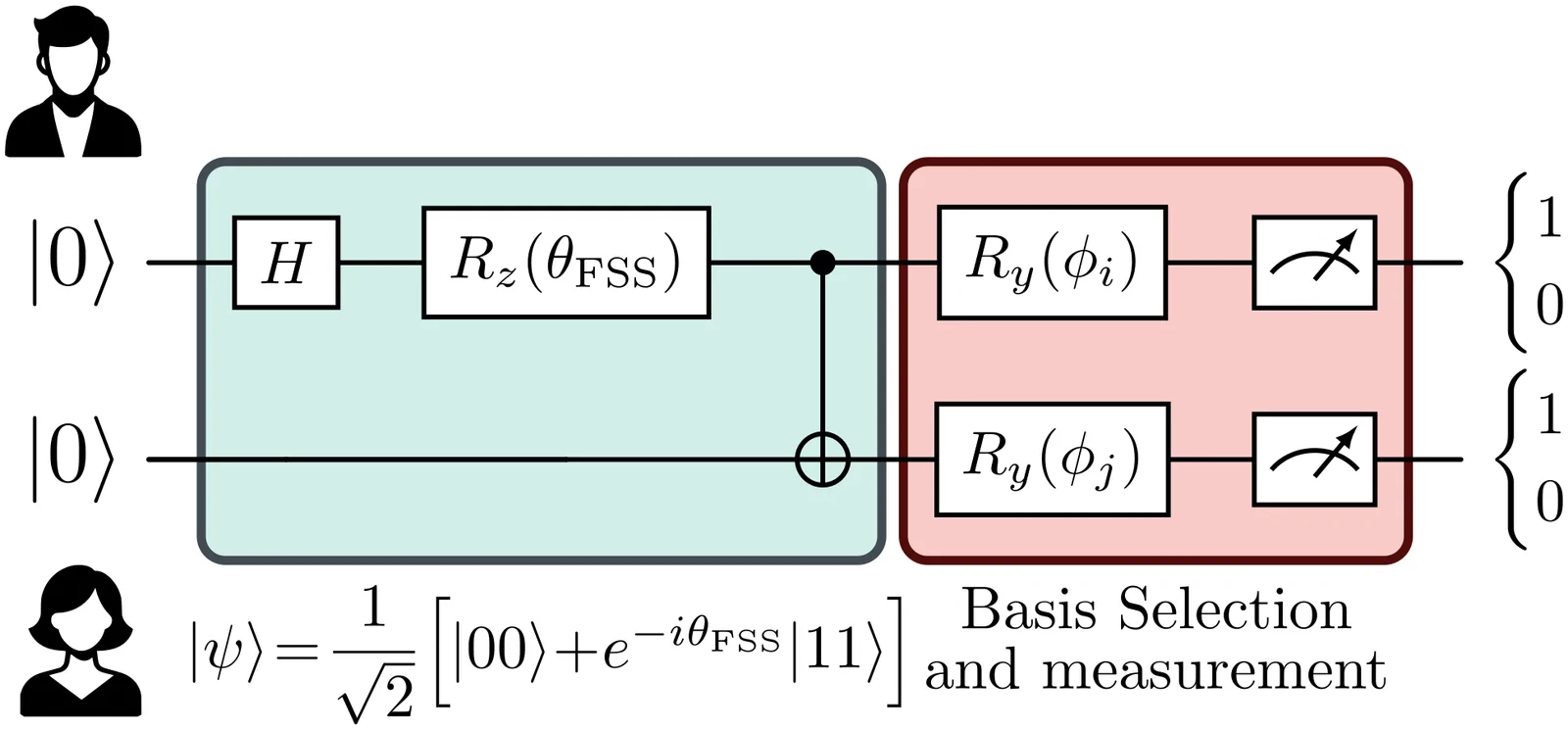
Quantum circuit for the transmission of a bit of information using the modified E91 scheme for a pure input state. In the green stage the dephased Bell state is created and in the pink stage Alice and Bob carry out the measurements along the directions $\phi_{i}$ and $\phi_{j}$, respectively, with *i* = 0,1,2 and *j* = *i* + 1.

The circuit starts by producing the dephased entangled state in terms of $\theta_{\text{FSS}}$ (green stage). Then introduces the random selection of the direction in which each participant carries out his/her measurement (pink stage). Event by event (associated to each entangled input state), they choose among their corresponding three available directions ($\phi_{i}$ with *i* = 0,1,2 for Alice and $\phi_{j}$ with *j* = 1,2,3 for Bob), which are defined in terms of the parameters α, and β, according to the orientations shown in Fig 2(b). The implementation of the latter involves a couple of Ry(−ϕℓ) rotation gates, that are respectively applied over the quantum channels to emulate the rotation of the polarization detectors, right before the associated measurements. Each measurement may yield either a 0 or a 1, which are correspondingly mapped to −1 or 1, in the language of equation 7.

Each of these events may become one of the bits in the key-string as long as the bases chosen by Alice and Bob coincide. Hence, to achieve a key with a number of bits long enough, the described process must be repeated a large number of times.

To evaluate the performance of the key distribution, we define the quantum computing-simulated total probability of correlation ($P_{\text{Corr}}^{QS}$) as the adequate metric. This is calculated as the ratio between the key-bits successfully transmitted (i.e., key-bits effectively correlated, yielding the same measurement for Alice and Bob in an event in which they chose coincident bases), and the ideally expected number of bits in the key (i.e., the number of events in which they chose coincident bases). Readily


$$\begin{array}{c} P_{\text{Corr}}^{QS}=\frac{\text{Number of correlated bits in the key-string}}{\text{Number of events measured in coincident bases}}. \end{array}$$ (15)

In the final stage of the computational implementation, the $P_{\text{Corr}}^{QS}$ value is registered as a function of the parameters α, β and $\theta_{\text{FSS}}$.

#### 4.1.2 Circuit for mixed input states.

If the input state is of the form shown in equation 3 instead of the one in equation 14, the quantum circuit is very similar to that used for pure states. However, it must include an additional preliminary stage in which the input state is selected between the dephased Bell’s state or one of the undesirable states, in such a way that any of the components of the statistical ensemble may go through the circuit and be subject of the execution of the protocol.

For the specific mixed state considered here, a random function chooses between the five involved components described in section 2.2, with probabilities *P*_ent_ = 0.9107, *P*_HH_ = 0.0030, *P*_HV_ = 0.0138, *P*_VH_ = 0.0278 and *P*_VV_ = 0.0447, respectively. Those particular values were taken from the experimentally reconstructed density operator reported in reference [15], and evidently satisfy equation 4.

After the random selector chooses one of the components, the protocol is executed analogously to the case for pure-states, by means of the same quantum circuit.

The definition of $P_{\text{Corr}}^{QS}$ is not modified by the mixed character of the input state, as compared to the case for pure input states. However, it is expected that its value becomes reduced as a consequence of the additional noise channel included in the stage of state preparation.

### 4.2 Connection between correlation probabilities and QBER

Correctness of the analytical model implies a match between $P_{\text{Corr}}^{T}$ and $P_{\text{Corr}}^{QS}$. To verify that, we must compare the analytical expression with the simulated results for different values of α, β and $\theta_{\text{FSS}}$.

The number of specific values of those angles for which it is adequate to carry out simulations that provide comparison points between the theoretical and computational correlation probabilities ($P_{\text{Corr}}^{T}$ and $P_{\text{Corr}}^{QS}$), is at principle arbitrary. It would depend on how dense one would prefer the grid to plot related quantities as functions of those angular parameters. The same cannot be said about the number of events for each set of angles *N*, which will be discussed below in section 5.1.

To make the results more understandable for readers used to quantum cryptography terminology, we can translate these correlation probabilities into the well-known quantum bit error rate (QBER), which is an important figure of merit for analyzing the performance of a QKD protocol [86,87]. It expresses the ratio between the discordant bits in the key and the total number of sifted bits (ideal extend of the key) [88].

Since QBER reflects how fallible a QKD process is, it tends to zero in idealized scenarios and increases as different noise channels and defects are included in the characterization of a protocol. In this case, where we only consider flaws in the entanglement source [phase i)], for the analytically derived (the quantum-computing simulated) correlation probability, it holds that


$$\begin{array}{c} {\text{QBER}}^{T(QS)}\;=\;1-P_{\text{Corr}}^{T(QS)}. \end{array}$$ (16)

If other faults were considered, their effects should be added and the QBER would be further increased.

Regarding eavesdropping, it is worth mentioning that the security benchmark in the protocol E91 is not based on the key but on measurements carried out along non-coincident bases. Hence, in this case the influence of espionage is not an extra factor contributing to the QBER, but a possibility that must be evaluated to decide if the key may be conserved or must be discarded [3].

Effects of phase and mixing in the input states on the security of the protocol, will be analyzed later in section 5.2.

## 5 Results

### 5.1 Convergence of $P_{Corr}^{QS}\to P_{Corr}^{T}$

To make the simulated data consistent with the expressions from the analytical approach, the number *N* must be large enough. This is because the theoretical prediction assumed a completely probabilistic behavior of the quantum system, that is strictly fulfilled only at the limit N→∞.

Then, to determine *N*, such that a satisfactory match between the theoretical predictions and the corresponding quantum computing-simulated values is obtained, we carried out a convergence test requiring that a discrepancy index ϵ be smaller than $2\times 10^{-3}$ for any tried set of angles.

Fig 4 shows the convergence tests for pure and mixed input states. The discrepancy index used there is calculated according to


$$\begin{array}{c} \epsilon =1-R^{2}=\frac{\sum_{n}{\left({P_{Corr}^{QS}}^{\left(n\right)}-{P_{Corr}^{T}}^{\left(n\right)}\right)}^{2}}{\sum_{n}{\left({P_{Corr}^{QS}}^{\left(n\right)}-\overset{―}{P_{Corr}^{QS}}\;\right)}^{2}}, \end{array}$$ (17)

where $\left\{{P_{Corr}^{QS}}^{\left(n\right)}\right\}$ stands for values computed based on equation 15, for a given *N* and all considered values of α, $\theta_{\text{FSS}}$, and β. $\overset{―}{P_{Corr}^{QS}}$ represents the mean value of the set $\left\{P_{Corr}^{QS^{\left(n\right)}}\right\}$. For each *N*, the plotted values of ϵ and their corresponding error bars (standard deviations), are calculated by averaging the values obtained for all possibilities of the angular parameters.

**Fig 4:**
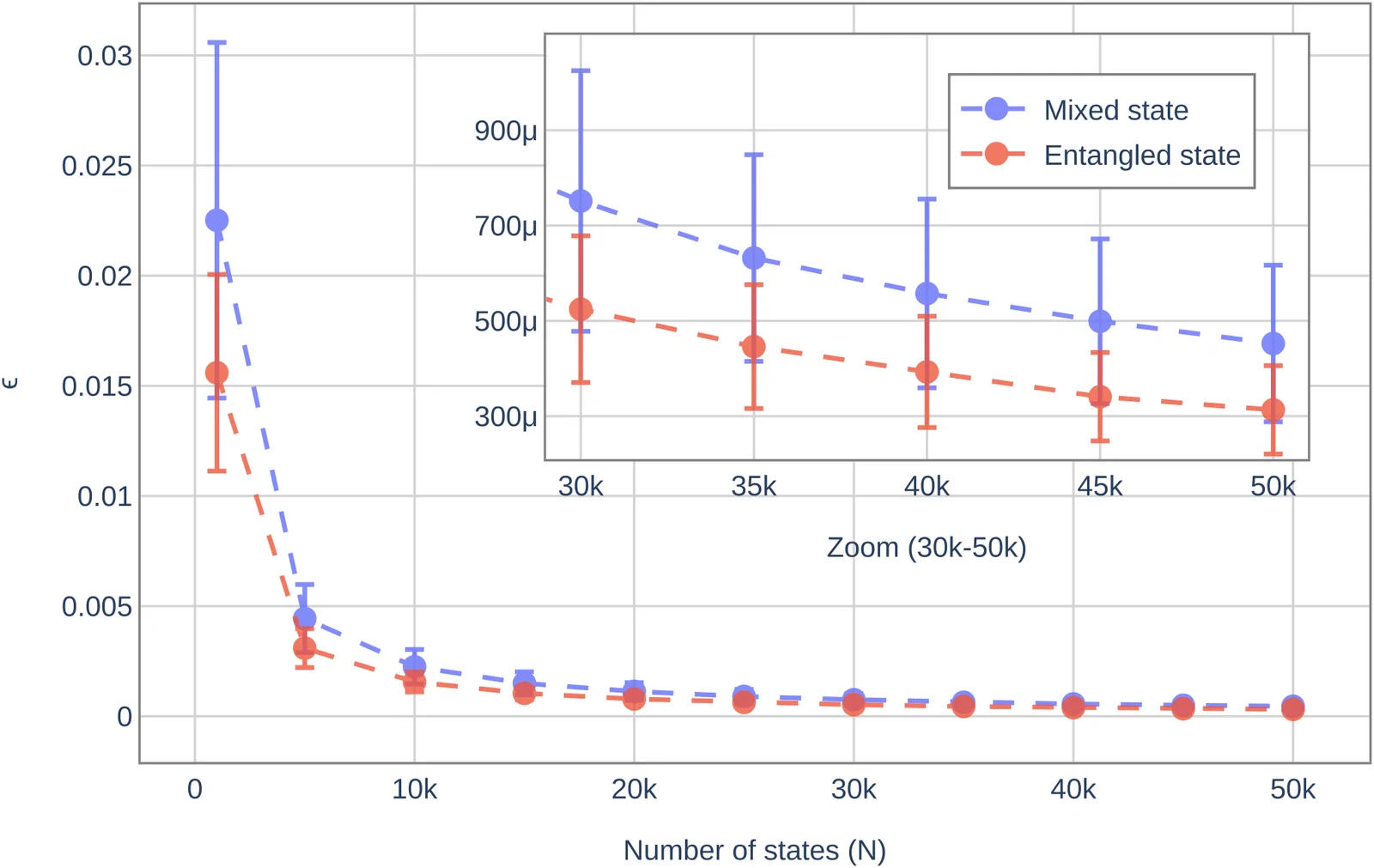
Convergence test for $P_{Corr}^{QS}\to P_{Corr}^{T}$. (Red) Pure entangled input states. (Blue) Mixed input states. The mixing parameters *P*_ent_ = 0.9107, $P_{HH}\approx 0.0030$, $P_{HV}\approx 0.0138$, $P_{VH}\approx 0.0278$ and $P_{VV}\approx 0.0447$, were used. The error bars indicate the corresponding standard deviations.

Such a convergence test led us to conclude that $N=2\times 10^{4}$ is sufficiently large for the required agreement. Nonetheless, the curves shown in the next section were obtained with $N=5\times 10^{4}$, in order to guarantee that a satisfactory match between the analytical model and the simulations is achieved.

### 5.2 Simulations and discussion

We execute the algorithm described in the previous section, to replicate the modified version of E91 protocol by means of the IBM’s Qiskit Aer simulator, which is a high performance framework for locally running quantum circuits [89]. It optimizes the computer’s resources toward efficient simulation of quantum computational calculations, with or without noise for the simulated gate operations. The machines used were: a desktop INTEL CORE I9-11900 5.2GHz with 16 cores and a RAM of 64GB, and a desktop INTEL CORE-ULTRA 9–258 5.8GHz with 24 cores, NVIDIA GeForce RTX 5090 with 32GB of video memory and RAM of 64GB.

We opted for executing those circuits in a quantum simulator instead of an actual quantum processor to avoid perturbations due to noise in phases ii) and iii) of the protocol, whose effects are not part of our model and have been partially studied in other works [52,53].

In the simulations, the parameters α and β were uniformly varied within the interval [0,π], while the studied range for $\theta_{\text{FSS}}$ was [0,2π]. For conciseness of the results, only six values were considered for α (i.e., ℓπ/8, ℓ=0,1,...,5). For each of the other angular parameters, we took 100 values, also uniformly spaced, for a total of $6\times 10^{4}$ points in the grid for either the pure and mixed input states.

As mentioned above in section 5.1, for each execution of the protocol, associated to a set of $\theta_{\text{FSS}}$, α, and β values, a total of $5\times 10^{4}$ events (entangled input states) were used to achieve satisfactory convergence (see Fig 4). Overall, we run $\sim 10^{10}$ times the designed quantum circuits.

Fig 5 shows the quantum-computational simulated $P_{\text{Corr}}^{QS}$ for pure input states, obtained for α=ℓπ/8 with ℓ=1,2,3,4, as functions of $\theta_{\text{FSS}}$ and β.

**Fig 5:**
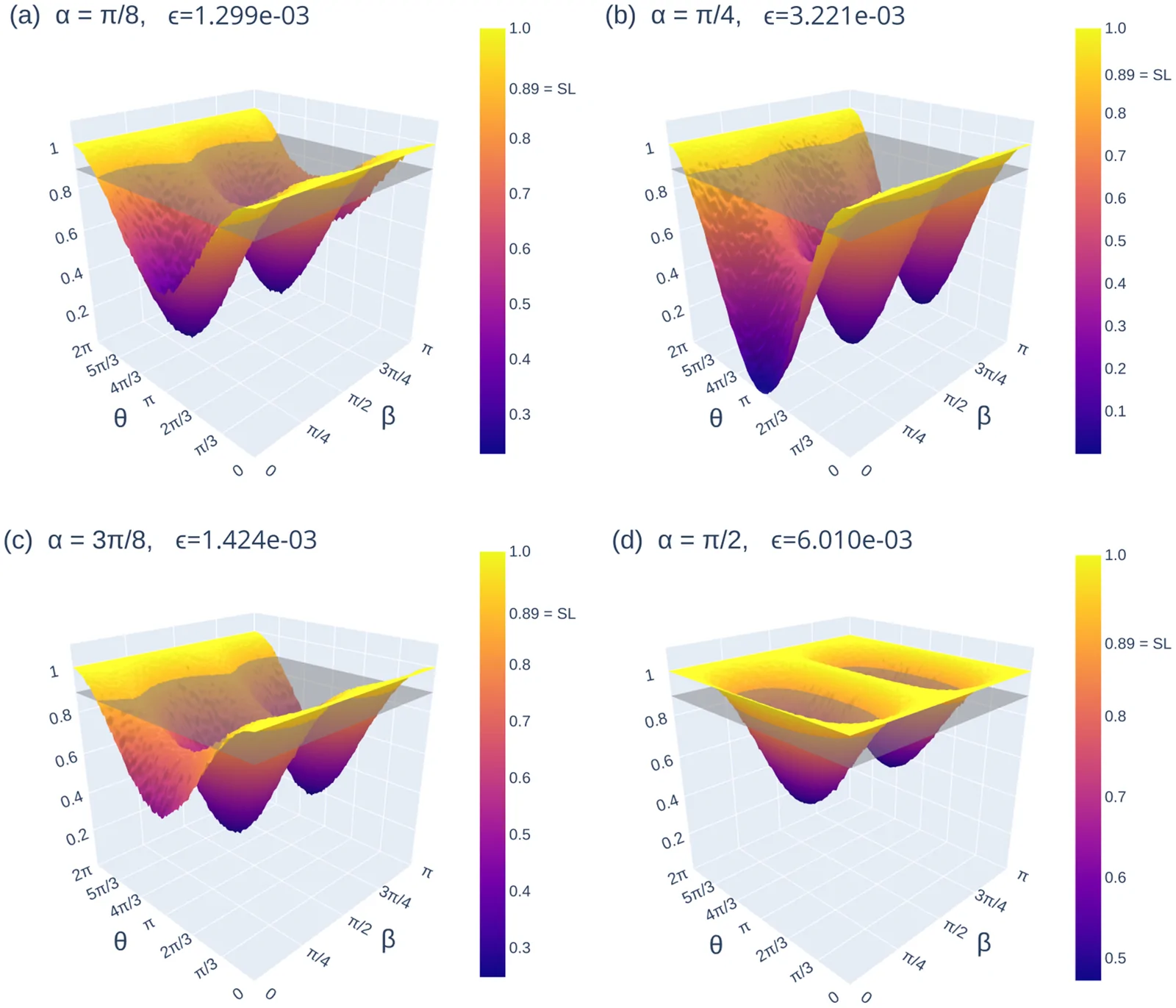
$P_{Corr}^{QS}$ for pure input states, with $\alpha =\ell \frac{\pi }{8}$ for ℓ=1,2,3,4 [(a), (b), (c) and (d)]. ϵ indicates the convergence index corresponding to each panel.

The plotted results correspond to the ones obtained with $N=5\times 10^{4}$, which guarantees quantitative agreement between $P_{\text{Corr}}^{QS}$ and $P_{\text{Corr}}^{T}$, according to the convergence test (see Fig 4).

Fig 6 also shows the quantum-computatio

nal simulated $P_{\text{Corr}}^{QS}$, but for mixed input states. The specific parameters used in the mixed states are the same as those used in Fig 4.

**Fig 6:**
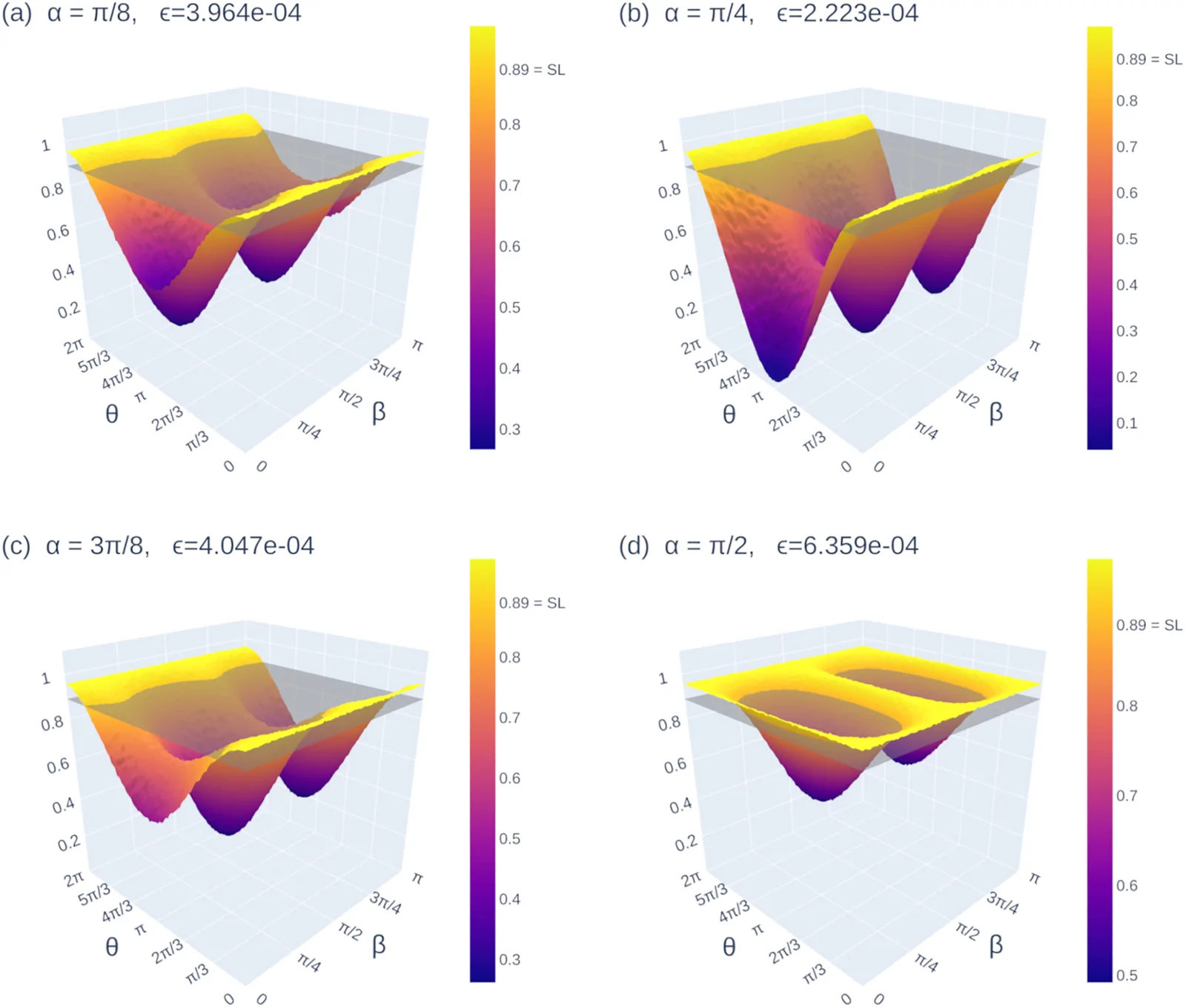
$P_{Corr}^{QS}$ for mixed input states, with $\alpha =\ell \frac{\pi }{8}$ for ℓ=1,2,3,4 [(a), (b), (c) and (d)]. ϵ indicates the convergence index corresponding to each panel.

These surface plots show a strong dependence of the correlation probability *P*_Corr_ (either of $P_{\text{Corr}}^{QS}$ or $P_{\text{Corr}}^{T}$) on $\theta_{\text{FSS}}$, for most values of β, presenting a recurrent minimum at $\theta_{\text{FSS}}=\pi$. The only exceptions to this behavior are observed in the cases α=nπ/2 with *n* integer, in which *P*_Corr_ is independent on $\theta_{\text{FSS}}$ for β=nπ/2. This is a trivial and useless scenario for which both polarizers are along the same direction, and that coincides with the orientation defined by the computational basis. In fact, any case in which β is a multiple of π, independently on the value of α, corresponds to a condition under which the protocol cannot be securely applied, because the randomness in the election of basis for measurements would be lost, and interception of Alice’s or Bob’s photons would result in exposition of the key.

It can be seen in Fig 5, how *P*_Corr_ oscillates on β with a period of π/2, and that the position and depth of the minima depend on α. Transversal cuts at different values of β have alike features in the considered interval. It initiates with *P*_Corr_ = 1, indicating an optimal performance of the protocol. Then, it decays until a minimum at $\theta_{\text{FSS}}=\pi$, where it starts increasing again until recovering ideal operation of the protocol at $\theta_{\text{FSS}}=2\pi$.

There are two scenarios in which the minimum *P*_Corr_ reaches extreme values. α=nπ/2 and α=(2n+1)π/4 (*n* = 0,1,2,3...).

In the former case, the minimum value for *P*_Corr_ is 0.5, indicating total randomness in the correlation of Alice’s and Bob’s measurements. In that configuration, the minima are located in β=(2n+1)π/4. Something expected, because orthogonality of the detection polarizers leads to accidental correlation [85]. Fig 7 (a) shows $P_{\text{Corr}}^{T}$ (solid line) for this particular case (α=π/8 and β=π/8). The corresponding quantum-computational simulated data $P_{\text{Corr}}^{QS}$ (black dots) are also shown to highlight the notorious coincidence between those correlations.

**Fig 7:**
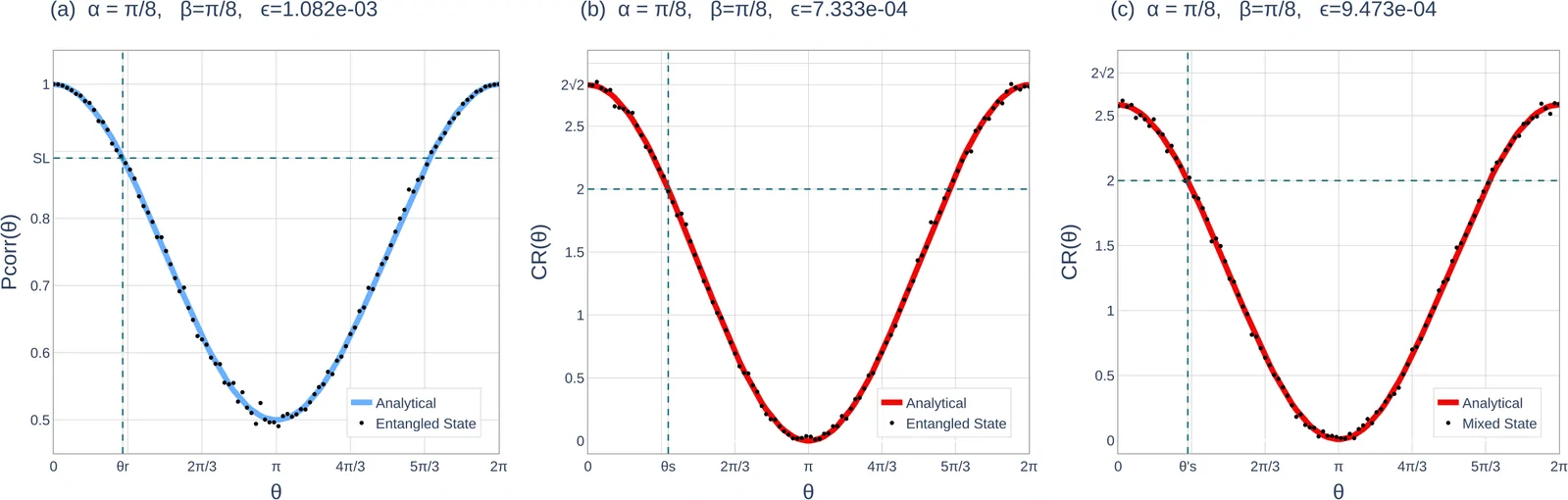
Ranges of key recoverability and transmission privacy. (a) $P_{\text{Corr}}^{T}$ (solid-blue line) and $P_{\text{Corr}}^{QS}$ (black dots) as functions of $\theta_{\text{FSS}}$. The vertical line indicates the limit of the region within which the key is recoverable. (b) Derived |CR|, from equation 9, as a function of $\theta_{\text{FSS}}$ (solid-red line), and the corresponding simulated data (black dots). The vertical line indicates the limit of the regions within which, violation of the Bell’s theorem guarantees privacy of the transmitted key. (c) As in (b), but for mixed input states. For all three panels α=β=π/8, and the correlation indexes between the analytical predictions and the simulations satisfy $\epsilon <2\times 10^{-3}$.

In the latter case, the lowest value for *P*_Corr_ is 0, which interestingly implies that in such configuration, for $\theta_{\text{FSS}}=\pi$, the intended correlation in Alice’s and Bob’s measurements, turns into perfect anticorrelation.

In all those plots, the horizontal planes indicate the threshold under which the transmitted key is not recoverable by means of error correction because its accuracy would lie below the Shannon limit (*QBER* < 0.11) [90,91].

Regarding privacy, since the |CR| quantity is essential for determining security of the protocol under potential presence of eavesdroppers, it is important to evaluate the validity of the corresponding derived expression, for which the data from the quantum-computational simulations can also be used. Particularly, the data from the cases in which non-coincident bases were chosen. Then, we analyze the simulated |CR| quantity in terms of $\theta_{\text{FSS}}$ to compare the results with the analytical expression.

Fig 7 (b) evidences the match between the formula in equation 9 (pure input states) and the data from the simulations for the case α=π/8 and β=π/8, i.e., the same orientations proposed originally by Ekert to maximize the violation of the Bell’s inequality CHSH. A high correlation between the predicted *CR* function and the results from the quantum computing implementation was found, with a correlation index of $\epsilon <2\times 10^{-3}$. Fig 7 (c) is similar to Fig 7 (b), but it allows comparison between equation 12 and the simulated data for mixed input states.

The oscillations of *CR* on the relative phase of the input state, imply that consulting such a quantity to prove security in the key transmission may trigger spurious alerts because |CR|≤2 could happen even in absence of eavesdropping. Fig 7 (b) [7 (c)] shows that for the particular angles α=β=π/8, if $\theta_{\text{FSS}}<\theta_{S}$ or $\left(2\pi +\theta_{S})>\theta_{\text{FSS}}>(2\pi -\theta_{S}\right)$ [$\theta_{\text{FSS}}<\theta_{S}^{\prime }$ or $\left(2\pi +\theta_{S}^{\prime })>\theta_{\text{FSS}}>(2\pi -\theta_{S}^{\prime }\right)$], the privacy criterion originally devised by Ekert for his protocol (|*CR*| > 2), can be applied.

Nevertheless, there is no need for such a criterion in the regions where QBER≥0.11, because disregarding whether the key is private or not, it cannot be recovered from the transmitted data. Comparison of the vertical lines in Fig 7 (a), (b) and (c), grants to conclude that for α=β=π/8, within the $\theta_{\text{FSS}}$ range where the protocol is usable, the security test is still applicable. For other values of α and β, Fig 5 in the supplementary material, show color plots on parameter surfaces where four different regions can be distinguished: i) Neither privacy can be guaranteed nor the key can be recovered (black areas), ii) Privacy can be guaranteed but the key cannot be recovered (red), iii) The key can be recovered but privacy cannot be guaranteed (yellow), and iv) Privacy can be guaranteed and the key cannot be recovered (green).

Summarizing, our analytical and computational results prove that the performance of the QKD protocol E91 depends substantially on the dephasing in the input entangled state. For a wide range of $\theta_{\text{FSS}}$ values, such a dephasing strongly diminishes the ability of the protocol for transmitting reliable keys. Further inclusion of the non-ideal purity of the entangled state, based on considerations about the realistic limitations of the QD photon source, yielded additional degrading of the transmitted key, reducing the $\theta_{\text{FSS}}$ range along which the protocol E91 is trustworthy.

Concerning proposals to overcome the negative effects of the phase and mixing in the input state, spectral filtering alongside effective reduction of either the FSS or the exciton lifetime in the QD used to produce the entangled states, appears as the most natural way of action. However, an interesting alternative emerging from the presented results, for mitigating the unfavorable effects of the dephasing, is to adjust the detection angles in the protocol implementation. According to Figs 5 and 6, α and β could be tuned to rise the minimum *P*_Corr_, extending the interval of $\theta_{\text{FSS}}$ values within which the protocol execution would yield recoverable keys (see Fig 5).

It is worth remarking that although these results were obtained considering the exciton FSS as the only cause of the phase in the entangled state produced by a QD source, they are applicable in situations where other sources of dephasing can be considered, e.g., valence band mixing or exciton-spin flipping by external fields [69].

As a final comment for this subsection, we would like to highlight that the validation of the analytical model by means of the quantum computational approach resulted much cheaper and faster than what the experimental counterpart would have been. These simulations were carried out in mid-scale desktop machines and the computing times were on the order of a few days.

### 5.3 Protocol BBM92 as a limit

The entanglement-based QKD protocol known as BBM92 [92], can be obtained as a particular case of the modified E91 scheme described in section 3, by setting α=0 and β=π/4 (see Fig 2(b)). It was devised by C. Bennett, G. Brassard and N. Mermin to securely transmit a key by using only two directions, corresponding to horizontal-vertical ({|H⟩,|V⟩}) and diagonal-antidiagonal ({|D⟩,|A⟩}) polarizations.

The BBM92 protocol has been chosen for QKD experimental implementations because of its simplicity, since it does not include Bell inequality measurements [93].

Inserting those specific values (0 and π/4) into equation 8, the corresponding correlation probability becomes


$$\begin{array}{c} P_{Corr}^{T}=\frac{3+C_{\theta_{\text{FSS}}}}{4}, \end{array}$$ (18)

which coincides with the previously reported expression for that protocol, in reference [47].

Nevertheless, the formulation developed in this work provides the possibility of enhancing the $P_{Corr}^{T}$ for a given value of $\theta_{\text{FSS}}$, by means of the observed β-dependence. Such correlation probability becomes


$$\begin{array}{c} P_{Corr}=\frac{1}{2}\left[1+C_{\beta }^{4}+S_{\beta }^{4}+2S_{\beta }^{2}C_{\beta }^{2}\;C_{\theta_{\text{FSS}}}\right], \end{array}$$ (19)

which reveals a mechanism to improve the efficacy of the BBM92 protocol under dephasing of the input state, by uplifting its minimum $P_{Corr}$ (see Fig 5 (d)).

The observed dependence on $\theta_{\text{FSS}}$ of the correlation probabilities of the protocols E91 and BBM92 is the same (equation 18), for the originally proposed angles in each case (α=β=π/8 for E91, and α=0 and β=π/4 for BBM92).

The privacy criteria are quite different for those protocols (Bell measurements for E91 vs comparison of parts of the shared key for BBM92). Eavesdropping is more easily detectable for E91 as long as it is executed in a $\theta_{\text{FSS}}$ range where the key is recoverable. However, the BBM92’s privacy criterion may be adopted by E91 to verify the key safety in ranges where the CHSH inequality is not a useful mechanism to detect espionage. Thus, considering the tunability of *P*_Corr_ through α and β, advantage may be taken of those zones where the key would be recoverable even if the Bell test is not useful (yellow regions in Fig 5).

## 6 Conclusions

We studied the performance of the QKD protocol E91 under the effects of dephased polarization-entangled states and mixing of emitted states, generated by radiative cascade in a QD source. We also investigated the influence of varying the orientation of the measurement polarizers by parameterizing the directions in which there can be coincidence of the analyzers.

We derived explicit expressions for the performance of the quantum distribution and for the quantity used to carry out the CHSH-type Bell test in the stage of security verification, as functions of the phase in the entangled state and of the varying angles in the protocol realization, in both scenarios pure and mixed input states. Those analytical expressions were satisfactorily validated by multiple simulations from a quantum computing implementation of the protocol, executed in the IBM’s Qiskit Aer simulator.

According to our results, the secret key rate for the transmission is substantially affected by the dephasing and impurity of the entangled state. Under some conditions, the value of this rate may be as low as 0.5, which corresponds to a scenario where the protocol becomes completely ineffective.

In turn, the parameter for the Bell test also oscillates on the magnitude of the dephasing, reaching values in which the CHSH inequality is not violated. This may lead to false positives in the stage of eavesdropping detection.

The observed dependence of the secret key rate on the varying angles of the measurement polarizers, suggest a mechanism to remediate the adverse effects generated on the reliability of the key transmission, either by the exciton fine structure splitting of the emitting quantum dot or by its reexcitation in the production of the two-photon input state.

These findings, that are extendable to the BBM92 protocol since it can be seen as a particular case within the presented formulation, provide valuable insight on standing challenges and potential solutions toward the large scale use of novel sources of entanglement in emerging technologies like quantum communication and quantum cryptography.

From a broader point of view, this work represents a vivid example of how currently available quantum computation tools are useful for emulating physical systems and processes whose experimental realization may turn arduous.

## S1 Appendix

### Calculation of the correlation probability for pure states

Let’s suppose that Alice and Bob respectively set their analyzers in the directions $\phi_{a}$ and $\phi_{b}$. The horizontal and vertical polarization eigenvalues (|H⟩ and |V⟩) are given by


$$\begin{array}{cc} \left|H\right\rangle & =C_{\phi_{\ell }}\left|{+}_{\phi_{\ell }}\right\rangle +S_{\phi_{\ell }}\left|{-}_{\phi_{\ell }}\right\rangle ,\\ \left|V\right\rangle & =-S_{\phi_{\ell }}\left|{+}_{\phi_{\ell }}\right\rangle +C_{\phi_{\ell }}\left|{-}_{\phi_{\ell }}\right\rangle , \end{array}$$ (20)

in terms of the corresponding Alice’s and Bob’s polarization eigenvalues $\left|\pm_{\phi_{\ell }}\right\rangle$, with ℓ=a,b [85].

Thus, the initial state of equation 2


$$\begin{array}{c} |\psi \rangle \;=\;\frac{1}{\sqrt{2}}\left[|H_{XX}H_{X}\rangle +e^{-i\theta_{\text{FSS}}}|V_{XX}V_{X}\rangle \right]. \end{array}$$ (21)

can be rewritten in the Alice’s and Bob’s basis, according to


$$\begin{array}{cc} \left|\psi \right\rangle =\frac{1}{\sqrt{2}}[ & \left|{+}_{\phi_{a}}{+}_{\phi_{b}}\right\rangle \left(C_{\phi_{a}}C_{\phi_{b}}+e^{-i\theta_{\text{FSS}}}S_{\phi_{a}}S_{\phi_{b}}\right)+\left|{-}_{\phi_{a}}{-}_{\phi_{b}}\right\rangle \left(S_{\phi_{a}}S_{\phi_{b}}+e^{-i\theta_{\text{FSS}}}C_{\phi_{a}}C_{\phi_{b}}\right)\\ + & \left|{+}_{\phi_{a}}{-}_{\phi_{b}}\right\rangle \left(C_{\phi_{a}}S_{\phi_{b}}-e^{-i\theta_{\text{FSS}}}S_{\phi_{a}}C_{\phi_{b}}\right)+\left|{-}_{\phi_{a}}{+}_{\phi_{b}}\right\rangle \left(S_{\phi_{a}}C_{\phi_{b}}-e^{-i\theta_{\text{FSS}}}C_{\phi_{a}}S_{\phi_{b}}\right)]. \end{array}$$ (22)

Because of orthogonality of the Alice’s and Bob’s bases, the probabilities of having correlated measurements, *P*_++_ and *P*_−−_, can now be straightforwardly obtained through the corresponding projections. Then


$$\begin{array}{cc} P_{++} & =|\langle {+}_{\phi_{a}},{+}_{\phi_{b}}|\psi \rangle {|}^{2},\\ & =\frac{1}{2}|C_{\phi_{a}}C_{\phi_{b}}+e^{-i\theta_{\text{FSS}}}S_{\phi_{a}}S_{\phi_{b}}{|}^{2},\\ & =\frac{1}{2}\left[C_{\phi_{a}}^{2}C_{\phi_{b}}^{2}+S_{\phi_{a}}^{2}S_{\phi_{b}}^{2}+2S_{\phi_{a}}C_{\phi_{a}}S_{\phi_{b}}C_{\phi_{b}}C_{\theta_{\text{FSS}}}\right]. \end{array}$$ (23)

Since the transmitted key is composed of measurements done in matching directions, we take $\phi_{a}=\phi_{b}=\phi$ and equation 23 becomes


$$\begin{array}{c} P_{++}=\frac{1}{2}\left[C_{\phi }^{4}+S_{\phi }^{4}+2S_{\phi }^{2}C_{\phi }^{2}C_{\theta_{\text{FSS}}}\right], \end{array}$$ (24)

inline with equation 7.

The calculation for *P*_−−_ is analogous and yields the same result.

The probability of having anticorrelated measurements, *P*_–+_ (which is the same as $P_{+-}$), is


$$\begin{array}{cc} P_{+-} & =|\langle \pm_{\phi_{a}},\mp_{\phi_{b}}|\psi \rangle {|}^{2},\\ & =\frac{1}{2}|C_{\phi_{a}}S_{\phi_{b}}-e^{-i\theta_{\text{FSS}}}S_{\phi_{a}}C_{\phi_{b}}{|}^{2},\\ & =\frac{1}{2}\left[C_{\phi_{a}}^{2}S_{\phi_{b}}^{2}+S_{\phi_{a}}^{2}C_{\phi_{b}}^{2}-2S_{\phi_{a}}C_{\phi_{a}}S_{\phi_{b}}C_{\phi_{b}}C_{\theta_{\text{FSS}}}\right]. \end{array}$$ (25)

### Calculation of the correlation probability for mixed states

The mixed state in equation 3, includes contributions from the basis states.

Analogously to the previous appendix section, once Alice (Bob) incoming particle has passed through the rotated polarizer, each possible uncorrelated state of the basis takes the form


$$\begin{array}{cc} \left|HH\right\rangle = & \left|{+}_{\phi_{a}}+\phi_{b}\right\rangle \left(C_{\phi_{a}}C_{\phi_{b}}\right)+\left|{-}_{\phi_{a}}-\phi_{b}\right\rangle \left(S_{\phi_{a}}S_{\phi_{b}}\right)+\\ & \left|{+}_{\phi_{a}}-\phi_{b}\right\rangle \left(C_{\phi_{a}}S_{\phi_{b}}\right)+\left|{-}_{\phi_{a}}+\phi_{b}\right\rangle \left(S_{\phi_{a}}C_{\phi_{b}}\right), \end{array}$$ (26)

$$\begin{array}{cc} \left|HV\right\rangle = & \left|{+}_{\phi_{a}}{+}_{\phi_{b}}\right\rangle \left(C_{\phi_{a}}S_{\phi_{b}}\right)-\left|{-}_{\phi_{a}}{-}_{\phi_{b}}\right\rangle \left(S_{\phi_{a}}C_{\phi_{b}}\right)-\\ & \left|{+}_{\phi_{a}}{-}_{\phi_{b}}\right\rangle \left(C_{\phi_{a}}C_{\phi_{b}}\right)+\left|{-}_{\phi_{a}}{+}_{\phi_{b}}\right\rangle \left(S_{\phi_{a}}S_{\phi_{b}}\right), \end{array}$$ (27)

$$\begin{array}{cc} \left|VH\right\rangle = & \left|{+}_{\phi_{a}}{+}_{\phi_{b}}\right\rangle \left(S_{\phi_{a}}C_{\phi_{b}}\right)-\left|{-}_{\phi_{a}}{-}_{\phi_{b}}\right\rangle \left(C_{\phi_{a}}S_{\phi_{b}}\right)+\\ & \left|{+}_{\phi_{a}}{-}_{\phi_{b}}\right\rangle \left(S_{\phi_{a}}S_{\phi_{b}}\right)-\left|{-}_{\phi_{a}}{+}_{\phi_{b}}\right\rangle \left(C_{\phi_{a}}C_{\phi_{b}}\right), \end{array}$$ (28)

$$\begin{array}{cc} \left|VV\right\rangle = & \left|{+}_{\phi_{a}}{+}_{\phi_{b}}\right\rangle \left(S_{\phi_{a}}S_{\phi_{b}}\right)+\left|{-}_{\phi_{a}}{-}_{\phi_{b}}\right\rangle \left(C_{\phi_{a}}C_{\phi_{b}}\right)-\\ & \left|{+}_{\phi_{a}}{-}_{\phi_{b}}\right\rangle \left(S_{\phi_{a}}C_{\phi_{b}}\right)-\left|{-}_{\phi_{a}}{+}_{\phi_{b}}\right\rangle \left(C_{\phi_{a}}S_{\phi_{b}}\right). \end{array}$$ (29)

In the initial basis, equation 3 takes the form


$$\begin{array}{cc} \rho =P_{\text{ent}}\left|\psi \right\rangle \left\langle \psi \right|+ & P_{HH}\left|HH\right\rangle \left\langle HH\right|+P_{HV}\left|HV\right\rangle \left\langle HV\right|+\\ & P_{VH}\left|VH\right\rangle \left\langle VH\right|+P_{VV}\left|VV\right\rangle \left\langle VV\right|. \end{array}$$ (30)

Based on this expression it is possible to compute the probability for Alice (Bob) to measure a + 1(+ 1) with the mixed state source. The $P_{++}^{M}$ from equation 23 takes the form


$$\begin{array}{cc} P_{++}^{M}= & P_{\text{ent}}{\left|\left\langle {+}_{\phi_{a}}{+}_{\phi_{b}}|\psi \right\rangle \right|}^{2}+P_{HH}{\left|\left\langle {+}_{\phi_{a}}{+}_{\phi_{b}}|HH\right\rangle \right|}^{2}+\\ & P_{HV}{\left|\left\langle {+}_{\phi_{a}}{+}_{\phi_{b}}|HV\right\rangle \right|}^{2}+P_{VH}{\left|\left\langle {+}_{\phi_{a}}{+}_{\phi_{b}}|VH\right\rangle \right|}^{2}+P_{VV}{\left|\left\langle {+}_{\phi_{a}}{+}_{\phi_{b}}|VV\right\rangle \right|}^{2},\\ = & \frac{P_{\text{ent}}}{2}\left[C_{\phi_{a}}^{2}C_{\phi_{b}}^{2}+S_{\phi_{a}}^{2}S_{\phi_{b}}^{2}+2S_{\phi_{a}}C_{\phi_{a}}S_{\phi_{b}}C_{\phi_{b}}C_{\theta_{\text{FSS}}}\right]+P_{HH}\left[C_{\phi_{a}}^{2}C_{\phi_{b}}^{2}\right]+\\ & P_{HV}\left[C_{\phi_{a}}^{2}S_{\phi_{b}}^{2}\right]+P_{VH}\left[S_{\phi_{a}}^{2}C_{\phi_{b}}^{2}\right]+P_{VV}\left[S_{\phi_{a}}^{2}S_{\phi_{b}}^{2}\right]. \end{array}$$ (31)

For $\phi_{a}=\phi_{b}=\phi$ the expression reduces to


$$\begin{array}{c} P_{++}^{M}=\frac{P_{\text{ent}}}{2}\left[C_{\phi }^{4}+S_{\phi }^{4}+2S_{\phi }^{2}C_{\phi }^{2}C_{\theta_{\text{FSS}}}\right]+P_{HH}C_{\phi }^{4}+P_{HV}C_{\phi }^{2}S_{\phi }^{2}+P_{VH}S_{\phi }^{2}C_{\phi }^{2}+P_{VV}S_{\phi }^{4}. \end{array}$$ (32)

The derivation for $P_{-\;\;-}^{M}$ is analogous, so that


$$\begin{array}{cc} P_{-\;\;-}^{M}= & \frac{P_{\text{ent}}}{2}\left[C_{\phi_{a}}^{2}C_{\phi_{b}}^{2}+S_{\phi_{a}}^{2}S_{\phi_{b}}^{2}+2S_{\phi_{a}}C_{\phi_{a}}S_{\phi_{b}}C_{\phi_{b}}C_{\theta_{\text{FSS}}}\right]+P_{HH}\left[S_{\phi_{a}}^{2}S_{\phi_{b}}^{2}\right]+\\ & P_{HV}\left[S_{\phi_{a}}^{2}C_{\phi_{b}}^{2}\right]+P_{VH}\left[C_{\phi_{a}}^{2}S_{\phi_{b}}^{2}\right]+P_{VV}\left[C_{\phi_{a}}^{2}C_{\phi_{b}}^{2}\right], \end{array}$$ (33)

and for coincident direction of polarizers


$$\begin{array}{cc} P_{-\;\;-}^{M}= & \frac{P_{\text{ent}}}{2}\left[C_{\phi }^{4}+S_{\phi }^{4}+2S_{\phi }^{2}C_{\phi }^{2}C_{\theta_{\text{FSS}}}\right]+P_{HH}\left[S_{\phi }^{4}\right]+\\ & \left(P_{HV}+P_{VH}\right)\left[C_{\phi }^{2}S_{\phi }^{2}\right]+P_{VV}\left[C_{\phi }^{4}\right]. \end{array}$$ (34)

The anticorrelated probabilities $P_{\pm \mp }^{M}$ may be also computed, yielding


$$\begin{array}{cc} P_{+-}^{M}= & \frac{P_{\text{ent}}}{2}\left[C_{\phi_{a}}^{2}S_{\phi_{b}}^{2}+S_{\phi_{a}}^{2}C_{\phi_{b}}^{2}-2S_{\phi_{a}}C_{\phi_{a}}S_{\phi_{b}}C_{\phi_{b}}C_{\theta_{\text{FSS}}}\right]+P_{HH}\left[C_{\phi_{a}}^{2}S_{\phi_{b}}^{2}\right]+\\ & P_{HV}\left[C_{\phi_{a}}^{2}C_{\phi_{b}}^{2}\right]+P_{VH}\left[S_{\phi_{a}}^{2}S_{\phi_{b}}^{2}\right]+P_{VV}\left[S_{\phi_{a}}^{2}C_{\phi_{b}}^{2}\right], \end{array}$$ (35)

$$\begin{array}{cc} P_{-+}^{M}= & \frac{P_{\text{ent}}}{2}\left[S_{\phi_{a}}^{2}C_{\phi_{b}}^{2}+C_{\phi_{a}}^{2}S_{\phi_{b}}^{2}-2S_{\phi_{a}}C_{\phi_{a}}S_{\phi_{b}}C_{\phi_{b}}C_{\theta_{\text{FSS}}}\right]+P_{HH}\left[S_{\phi_{a}}^{2}C_{\phi_{b}}^{2}\right]+\\ & P_{HV}\left[S_{\phi_{a}}^{2}S_{\phi_{b}}^{2}\right]+P_{VH}\left[C_{\phi_{a}}^{2}C_{\phi_{b}}^{2}\right]+P_{VV}\left[C_{\phi_{a}}^{2}S_{\phi_{b}}^{2}\right]. \end{array}$$ (36)

## S2 Appendix

### Derivation of the correlation probability for the modified protocol

The probability of getting correlated measurements in an event in which the bases chosen by Alice and Bob coincide, even if none of those bases correspond to the vertical-horizontal one, is the addition of the probability when both participants chose $\phi_{1}$ plus the probability when they choose $\phi_{2}$, namely


$$\begin{array}{cc} P_{\text{Corr}}\;= & \;P_{\phi_{1}}\left[P_{\pm \pm }(\phi_{1},\theta_{\text{FSS}})\right]+P_{\phi_{2}}\left[P_{\pm \pm }(\phi_{2},\theta_{\text{FSS}})\right], \end{array}$$ (37)

where $P_{\phi_{i}}$ for *i* = 1,2 is the probability of choosing $\phi_{i}$ as the measurement orientation in a coincident event. Since the election of basis before each measurement is random, then $P_{\phi_{1}}=P_{\phi_{2}}=\frac{1}{2}$. Then


$$\begin{array}{cc} P_{\text{Corr}}\;= & \frac{1}{2}\left[P_{\pm \pm }(\phi_{1},\theta_{\text{FSS}})+P_{\pm \pm }(\phi_{2},\theta_{\text{FSS}})\right]. \end{array}$$ (38)

In the optical implementation of the original protocol ($\phi_{1}=\pi /8$ and $\phi_{2}=\pi /4$), this total probability turns into


$$\begin{array}{cc} P_{\text{Corr}}\;= & \frac{5+3C_{\theta_{\text{FSS}}}}{8}. \end{array}$$ (39)

As expected, if the FSS vanishes *P*_Corr_ = 1, and the protocol would work optimally in absence of eavesdropping.

For the more general case of the modified protocol, in which the angles defining $\phi_{1}$ and $\phi_{2}$ are variable, the total probability of correlation for measurements along the coincident orientations reads


$$\begin{array}{cc} P_{\text{Corr}}\; & =\;\frac{1}{2}\left[P_{\pm \pm }(\alpha ,\theta_{\text{FSS}})+P_{\pm \pm }(\alpha +\beta ,\theta_{\text{FSS}})\right],\\ & =\frac{1}{2}[C_{\alpha }^{4}+S_{\alpha }^{4}+2S_{\alpha }^{2}C_{\alpha }^{2}C_{\theta_{\text{FSS}}}+\;C_{\alpha +\beta }^{4}+S_{\alpha +\beta }^{4}+2S_{\alpha +\beta }^{2}C_{\alpha +\beta }^{2}C_{\theta_{\text{FSS}}}]. \end{array}$$ (40)

## S3 Appendix

### Derivation of the angle-dependent *CR* quantity

Now, we can use the probabilities of correlated and anticorrelated measurements to obtain the correlation coefficients $E_{ab}\equiv E(\phi_{a},\phi_{b},\theta_{\text{FSS}})$, where $\phi_{a}$ ($\phi_{b}$) is the direction selected by Alice (Bob). Those coefficients are given by [19]


$$\begin{array}{c} E_{ab}=P_{++}+P_{-\;\;-}-P_{+-}-P_{-+}. \end{array}$$ (41)

Thus, inserting equations 23 and 25 into equation 41, the correlation coefficients turn into


$$\begin{array}{cc} E_{ab}= & C_{\phi_{a}}^{2}C_{\phi_{b}}^{2}+S_{\phi_{a}}^{2}S_{\phi_{b}}^{2}+2S_{\phi_{a}}C_{\phi_{a}}S_{\phi_{b}}C_{\phi_{b}}C_{\theta_{\text{FSS}}}\\ & -C_{\phi_{a}}^{2}S_{\phi_{b}}^{2}-S_{\phi_{a}}^{2}C_{\phi_{b}}^{2}+2S_{\phi_{a}}C_{\phi_{a}}S_{\phi_{b}}C_{\phi_{b}}C_{\theta_{\text{FSS}}},\\ = & C_{\phi_{a}}^{2}\left[C_{\phi_{b}}^{2}-S_{\phi_{b}}^{2}\right]-S_{\phi_{a}}^{2}\left[C_{\phi_{b}}^{2}-S_{\phi_{b}}^{2}\right]+4S_{\phi_{a}}C_{\phi_{a}}S_{\phi_{b}}C_{\phi_{b}}C_{\theta_{\text{FSS}}},\\ = & \left[C_{\phi_{a}}^{2}-S_{\phi_{a}}^{2}\right]C_{2\phi_{b}}+S_{2\phi_{a}}S_{2\phi_{b}}C_{\theta_{\text{FSS}}},\\ = & C_{2\phi_{a}}C_{2\phi_{b}}+S_{2\phi_{a}}S_{2\phi_{b}}C_{\theta_{\text{FSS}}}. \end{array}$$ (42)

Hence, the *CR* quantity to test the CHSH inequality is given by


$$\begin{array}{cc} CR= & E_{01}+E_{23}-E_{03}+E_{21},\\ = & C_{2\phi_{0}}C_{2\phi_{1}}+S_{2\phi_{0}}S_{2\phi_{1}}C_{\theta_{\text{FSS}}}+C_{2\phi_{2}}C_{2\phi_{3}}+S_{2\phi_{2}}S_{2\phi_{3}}C_{\theta_{\text{FSS}}}\\ & -C_{2\phi_{0}}C_{2\phi_{3}}-S_{2\phi_{0}}S_{2\phi_{3}}C_{\theta_{\text{FSS}}}+C_{2\phi_{2}}C_{2\phi_{1}}+S_{2\phi_{2}}S_{2\phi_{1}}C_{\theta_{\text{FSS}}},\\ = & C_{\theta_{\text{FSS}}}[S_{2\phi_{0}}\left(S_{2\alpha }-S_{2\phi_{3}}\right)+S_{2(\alpha +\beta )}\left(S_{2\phi_{3}}+S_{2\alpha }\right)]\\ & +C_{2\phi_{0}}\left(C_{2\alpha }-C_{2\phi_{3}}\right)+C_{2(\alpha +\beta )}\left(C_{2\phi_{3}}+C_{2\alpha }\right), \end{array}$$ (43)

in which substitution of the values $\phi_{0}=0$ and $\phi_{3}=3\pi /8$ leads to


$$\begin{array}{cc} CR= & C_{\theta_{\text{FSS}}}S_{2(\alpha +\beta )}\left[\frac{1}{\sqrt{2}}+S_{2\alpha }\right]+C_{2(\alpha +\beta )}\left[C_{2\alpha }-\frac{1}{\sqrt{2}}\right]+C_{2\alpha }+\frac{1}{\sqrt{2}}, \end{array}$$ (44)

that corresponds to equation 9.

### Derivation of the angle-dependent *CR* quantity for mixed states

The start point is the expression of the equation 41, in terms of the correlation and anticorrelation probabilities for mixed states given by the equations 31, 33, 35 and 36. Then, the additional components in the mixed ensemble must be included, according to


$$\begin{array}{cc} E_{ab}^{M}=\; & P_{\text{ent}}\left(C_{2\phi_{a}}C_{2\phi_{b}}+S_{2\phi_{a}}S_{2\phi_{b}}C_{\theta_{\text{FSS}}}\right)+P_{HH}\left[C_{2\phi_{a}}C_{2\phi_{b}}\right]+\\ & P_{VV}\left[C_{2\phi_{a}}C_{2\phi_{b}}\right]-P_{HV}\left[C_{2\phi_{a}}C_{2\phi_{b}}\right]-P_{VH}\left[C_{2\phi_{a}}C_{2\phi_{b}}\right],\\ =\; & P_{\text{ent}}\left(C_{2\phi_{a}}C_{2\phi_{b}}+S_{2\phi_{a}}S_{2\phi_{b}}C_{\theta_{\text{FSS}}}\right)+\\ & \left(P_{HH}+P_{VV}-P_{HV}-P_{VH}\right)C_{2\phi_{a}}C_{2\phi_{b}}. \end{array}$$ (45)

subsequently, equation 43 takes the form


$$\begin{array}{cc} CR^{M}= & P_{\text{ent}}(C_{\theta_{\text{FSS}}}\left[S_{2\phi_{0}}\left(S_{2\phi_{1}}-S_{2\phi_{3}}\right)+S_{2\phi_{2}}\left(S_{2\phi_{3}}+S_{2\phi_{1}}\right)\right]\\ & +C_{2\phi_{0}}\left(C_{2\phi_{1}}-C_{2\phi_{3}}\right)+C_{2\phi_{2}}\left(C_{2\phi_{3}}+C_{2\phi_{1}}\right))\\ & +\left(P_{HH}+P_{VV}-P_{HV}-P_{VH}\right)(C_{2\phi_{0}}\left(C_{2\phi_{1}}-C_{2\phi_{3}}\right)+C_{2\phi_{2}}\left(C_{2\phi_{3}}+C_{2\phi_{1}}\right)). \end{array}$$ (46)

By substituting $\phi_{0}=0$, $\phi_{1}=\alpha$, $\phi_{2}=\alpha +\beta$, $\phi_{3}=3\pi /8$ the equation 46 takes the form


$$\begin{array}{cc} CR^{M}= & \left(P_{\text{ent}}+P_{HH}+P_{VV}-P_{HV}-P_{VH}\right)\left[C_{2\phi_{0}}\left(C_{2\phi_{1}}-C_{2\phi_{3}}\right)+C_{2\phi_{2}}\left(C_{2\phi_{3}}+C_{2\phi_{1}}\right)\right]+\\ & P_{\text{ent}}C_{\theta_{\text{FSS}}}\left[S_{2\phi_{0}}\left(S_{2\phi_{1}}-S_{2\phi_{3}}\right)+S_{2\phi_{2}}\left(S_{2\phi_{3}}-S_{2\phi_{1}}\right)\right] \end{array}$$ (47)

## Supporting information

S1 FigInteractive figure.This figure eases visualization of $P_{Corr}^{T}$ and |*CR*| as functions of $\theta_{\text{FSS}}$ and β (α=π/8) for pure input states.(HTML)

S2 FigRanges of recoverability of the transmitted key vs ranges of privacy verifiability.The curves show the values $\theta_{C}$ and $\theta_{S}$ for $\alpha =\ell \frac{\pi }{8}$, with ℓ=1,2,3,4 [(a), (b), (c) and (d)].(TIFF)

S1 File Data from the simulations and code. Here is the link to the data from the quantum-computational simulations for both cases, pure and mixed input states. Also for the code used to run the simulations in Qiskit.
(ZIP)
